# Supplementary material for: A chromosome-level assembly of the pine processionary moth (Thaumetopoea pityocampa) genome
Source: J Hered. 2026 Jun 11;117(6):1392–405. doi: 10.1093/jhered/esag035 (PMC13733544; doi:10.1093/jhered/esag035)
Supplement: Supplementary file 1 [file supplementary_material_esag035.zip › media-2_esag035.pdf]

Supplementary Tables

	GridION Run (Q>9)	Promethion Run (Q>9)
**SRA accession ID**	SRR32477507	SRR32477506
**Nb. reads**	39,972 (16,783)	6,945,559 (5,055,546)
**Total yield in Gbp**	0.513 (0.249)	102.4 (81.54)
**Average read length in bp**	12,845 (14,825)	14,750 (16,130)
**N50 read length in bp)**	21,316 (21,428)	22,070 (22,271)
**Longest read in bp**	103,053 (103,053)	1,149,683 (157,788)
**Mean Q score**	7.89 (10.1)	10.2 (11.8)
**% reads with Q>10**	21.1 (50.3)	64.7 (88.9)
**Nb of active pores**	88 (73)	2,705 (2,684)
**Coverage (with GS=616 Mb)**	0.83 (0.40)	166.3 (132.4)

Table S1: Summary statistics for the long reads obtained with the two ONT sequencing runs. Metrics are reported for either all reads or considering only reads with a quality score Q>9 (in parentheses).

ID	Sex	SRA ID	Mreads (Gb) after filtering	% duplicate (`fastp`)	Mreads mapped (% prop. paired)	Realized median coverage
SP-M1	M	SRR32477503	149.26 (18.6)	1.35	132.6 (97.4)	28
SP-M2	M	SRR32477502	173.82 (21.7)	1.25	154.3 (97.5)	33
SP-F3	F	SRR32477501	155.71 (19.5)	1.39	135.2 (97.4)	29
SP-F4	F	SRR32477500	156.60 (19.6)	1.52	135.5 (97.3)	29

Table S2: Summary statistics for the short-read sequence data generated for the two males and females used for the identification of sex-linked and autosomal scaffolds.

Chromosome	Nb. SNPs	Physical Lengh in Mb (% covered)	Genetic Lengh in cM	cM to Mb
1	33	25.8 (97.1)	80.2	3.11
2	28	24.6 (96.7)	71.9	2.92
3	25	19.8 (92.8)	62.5	3.16
4	21	16.9 (85.5)	33.3	1.98
5	17	15.0 (90.7)	70.8	4.73
6	12	14.9 (91.2)	43.8	2.93
7	15	9.32 (67.2)	31.2	3.35
8	15	12.5 (90.6)	50.0	3.98
9	6	10.5 (78.4)	32.3	3.08
10	12	12.5 (94.2)	36.5	2.92
11	14	12.1 (93.7)	83.3	6.86
12	17	12.4 (96.6)	45.8	3.68
13	11	12.3 (96.1)	75.0	6.12
14	16	12.3 (96.9)	45.8	3.72
15	13	11.6 (93.3)	58.3	5.02
16	12	9.09 (73.4)	57.3	6.30
17	9	11.2 (90.4)	57.3	5.13
18	13	9.73 (79.7)	42.7	4.39
19	18	11.7 (96.4)	68.8	5.86
20	12	8.90 (76.3)	52.1	5.85
21	19	11.2 (96.8)	71.9	6.41
22	15	9.56 (82.9)	54.2	5.67
23	15	10.1 (88.2)	83.3	8.26
24	14	10.6 (92.5)	53.1	5.02
25	15	10.5 (94.6)	54.2	5.18
26	12	7.53 (68.6)	28.1	3.73
27	7	7.64 (70.2)	27.1	3.54
28	13	10.5 (97.3)	38.5	3.66
29	19	9.88 (91.4)	77.1	7.80
30	12	10.5 (97.4)	44.8	4.26
31	15	9.74 (91.6)	54.2	5.56
32	12	9.40 (92.3)	45.8	4.88
33	19	8.76 (87.8)	54.2	6.18
34	11	6.85 (69.9)	70.8	10.3
35	9	7.58 (77.4)	45.8	6.05
36	18	8.78 (94.5)	53.1	6.05
37	10	8.75 (94.3)	49.0	5.59
38	18	8.18 (89.8)	58.3	7.13
39	9	5.90 (64.7)	49.0	8.30
40	14	8.43 (93.4)	39.6	4.69
41	9	7.58 (84.6)	47.9	6.32
42	9	7.49 (87.6)	49.0	6.54
43	9	6.59 (77.6)	59.4	9.01
44	8	7.32 (87.4)	35.4	4.84
45	6	5.72 (76.8)	61.5	10.7
46	6	7.16 (96.7)	63.5	8.88
47	9	5.17 (79.7)	37.5	7.25
48	8	1.07 (18.3)	8.33	7.81
49	13	3.77 (67.0)	43.8	11.6
ALL	672	505.4 (87.2)	2557.3	5.06

Table S3: Comparison between the physical and genetic maps of the autosomes. For each chromosome, the table lists the number of markers (from collapsed RAD loci; see main text), the physical length in megabases (Mb) corresponding to the distance between the first and last marker on the `Tpit_2.1` assembly (with the percentage of chromosome coverage in parentheses), the estimated genetic length in centimorgans (cM), and the ratio of genetic to physical distance (cM/Mb). The last row provides cumulative estimates across all chromosomes. Genetic distances were computed according to the order of the assembly, assuming no recombination in females, and using the Morgan (linear) mapping function (Rastas, 2017).

**TE Classification**	**Coverage (in bp)**	**Copy Number**	**Genome Coverage (in %)**	**TE Family Count**
DNA	52,179,497	154,290	8.47	327
Rolling Circle	49,056,372	166,094	7.97	75
Penelope	505,978	1,850	0.0822	4
LINE	77,212,988	237,713	12.5	296
SINE	2,608,800	14,533	0.424	6
LTR	19,417,146	18,904	3.15	149
Other (Simple Repeat, Microsatellite, RNA)	4,349,319	19,243	0.706	1,037
Unclassified	102,149,994	425,566	16.6	1,147
Non-Repeat	308,368,143	–	50.1	–

Table S4: Summary of TE content in the *T. pityocampa* genome based on `EarlGrey` (v6.0.1; Baril *et al.*, 2024) classification. Genomic coverage is expressed as a percentage of the total `Tpit_2.1` assembly (615.8 Mb).

Gene (UniProt ID) Organism	*T. pityocampa* Gene ID (position)	BLAST align. length in bp (% id.; e-val)	Blast2GO annotation
timeless (Q3ZTQ6) *Danaus plexippus*	g268 1:11852874-11871503	1,274 (61.5; 0)	timeless
CRY-2 (Q0QWP3) *Danaus plexippus*	g3215 2:12457997-12482032	759 (69.2; 0)	cryptochrome-1-like
vrille (A0A4Y1PAQ0) *Helicoverpa armigera*	g718 10:8958265-8959359	367 (89.4; 0)	WASH complex subunit
FBXL (Q9VY46) *Drosophila melanogaster*	g1639 14:6461689-6487288	605 (37.7; $< 10^{-128}$)	F-box LRR-repeat protein
CRY-1 (O77059) *Drosophila melanogaster*	g3614 20:3381745-3398079	535 (55.9; 0)	cryptochrome-1
clockwork (A0A8R2G768) *Bombyx mori*	g3864 21:3758674-3805850	448 (76.6; 0)	transcription factor cwo
doubletime (O76324) *Drosophila melanogaster*	g4464 23:10533610-10546016	299 (85.0; 0)	casein kinase I
jetlag (Q0E8T8) *Drosophila melanogaster*	g5130 26:9184097-9185514	255 (38.4; $< 10^{-61}$)	F-box/ LRR-repeat protein
Abl, dash (P00522) *Drosophila melanogaster*	g5445 28:2355794-2472236	443 (49.0; $< 10^{-129}$)	tyrosine-protein kinase Src42A
shaggy (J7G1D3) *Biston betularia*	g6509 31:622903-686762	272 (98.5; 0)	glycogen synthase kinase-3 beta
slimb (A0A212FH45) *Danaus plexippus*	g8084 38:2271243-2289937	490 (94.7; 0)	beta-TrCP
neurl4 (A0A1L4AAD6) *Drosophila melanogaster*	g11304 Z:14329649-14371365	1,133 (43.6; 0)	neuralized-like protein 4
nep2 (A0A0B4K692) *Drosophila melanogaster*	g11305 Z:14376632-14445049	771 (54.9; 0)	neprilysin-2
clock (Q6VRU6) *Antheraea pernyi*	g11362 Z:16936019-16961825	567 (66.0; 0)	circadian locomoter output cycles protein kaput
period (Q58A64) *Bombyx mori*	g11389 Z:17953375-17989958	968 (58.1; 0)	period
pdp1 (Q8SZT1) *Drosophila melanogaster*	g11506 Z:24582256-24733232	241 (71.0; $< 10^{-107}$)	Not annotated
bmal1, cycle (O61734) *Drosophila melanogaster*	g11509 Z:24775996-24876815	404 (49.0; $< 10^{-125}$)	cycle

Table S5: Manual characterization of known Lepidopteran circadian genes in the *T. pityocampa* annotation. Homologous protein sequences were recovered from UniProt (within Lepidoptera when available, or using *D. melanogaster* if not) and blasted against the *T. pityocampa* proteome. Best hits are given with their genomic location and their Blast2GO annotation result.

Organism (Genome version)	RefSeq or BIPAA ID (URL)
Bombyx mori (ASM3026992v2)	GCF_030269925.1 (https://www.ncbi.nlm.nih.gov/datasets/genome/GCF_030269925.1/)
Drymonia ruficornis (ilDryRufi1.1)	GCA_947859195.1 (https://www.ncbi.nlm.nih.gov/datasets/genome/GCA_947859195.1/)
Notodonta ziczac (ilNotZicz1.1)	GCA_918843915.1 (https://www.ncbi.nlm.nih.gov/datasets/genome/GCA_918843915.1/)
Pheosia tremula (ilPheTrem1.1)	GCA_905333125.1 (https://www.ncbi.nlm.nih.gov/datasets/genome/GCA_905333125.1/)
Ptilodon capucinus (ilPtiCapc1.1)	GCA_914767695.1 (https://www.ncbi.nlm.nih.gov/datasets/genome/GCA_914767695.1/)
Spodoptera frugiperda (7.1)	OGS7.1_20230705 (https://bipaa.genouest.org/sp/spodoptera_frugiperda_corn/download/spodoptera_frugiperda_corn/assembly_7.0/annotation_OGS7.1/)

Table S6: List of the genomes used for the comparative genomics analysis

**Gene family**	***T. pityocampa***	***H. armigera***	***B. mori***	***S. frugiperda***
**CYP**	78	112	83	122
**CCE**	56	97	78	96
**GST**	30	47	25	41
**UGT**	23	41	43	40
**ABC**	49	54	54	67
**Total**	236	351	283	366

Table S7: Detoxification genes annotated in the genome of *T. pityocampa* and comparison with other Leipdoptera species.

Supplementary Figures

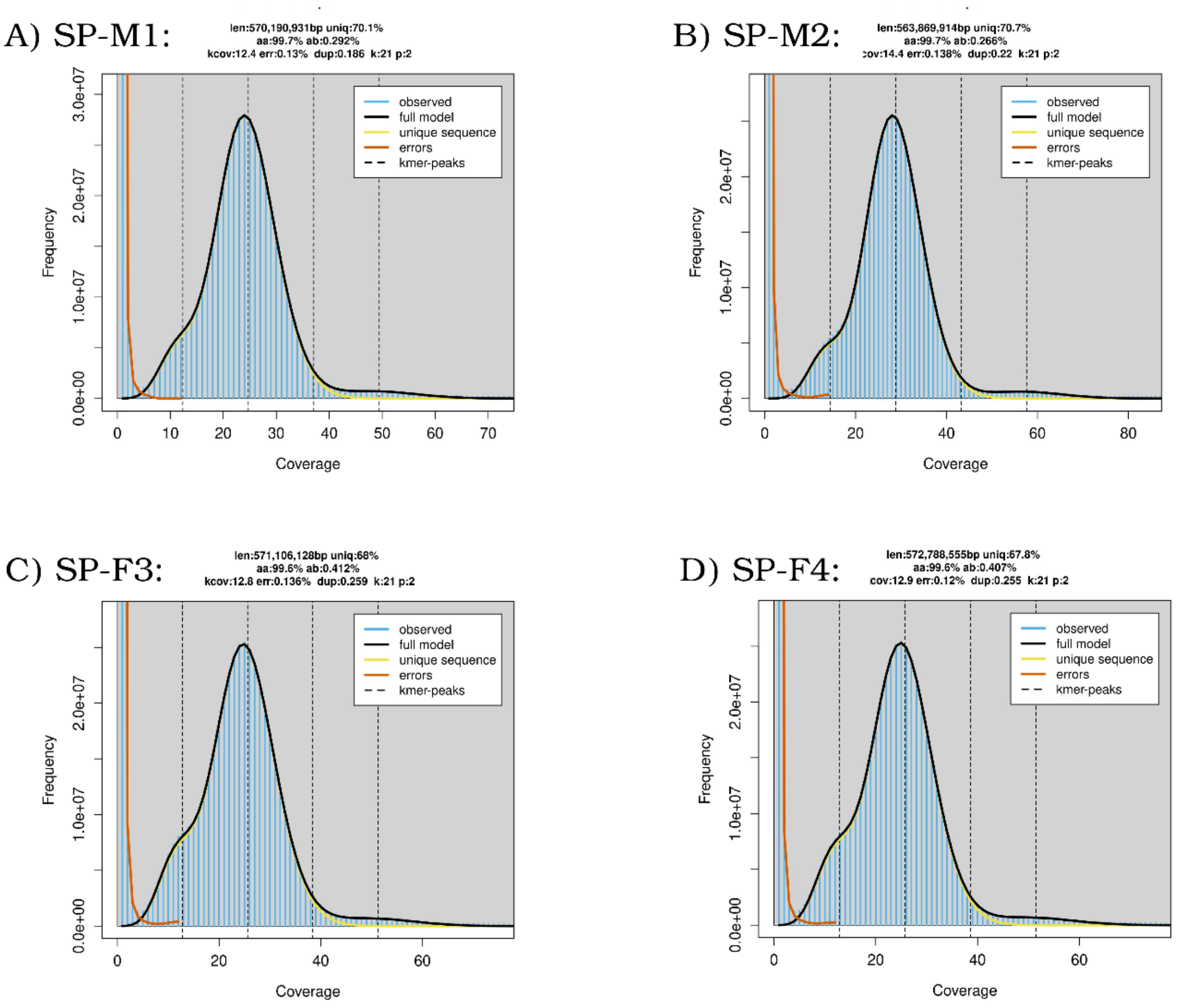

Figure S1: Estimates of the haploid genome sizes short read Whole-Genome Sequence data available for two males and two females sampled in Leiria (Portugal). The estimates were obtained using `GenomeScope` (v2.0; Ranallo-Benavidez *et al.*, 2020) from the modeling of the k-mer spectrum (with k=21) computed with `JellyFish` (Marçais and Kingsford, 2011).

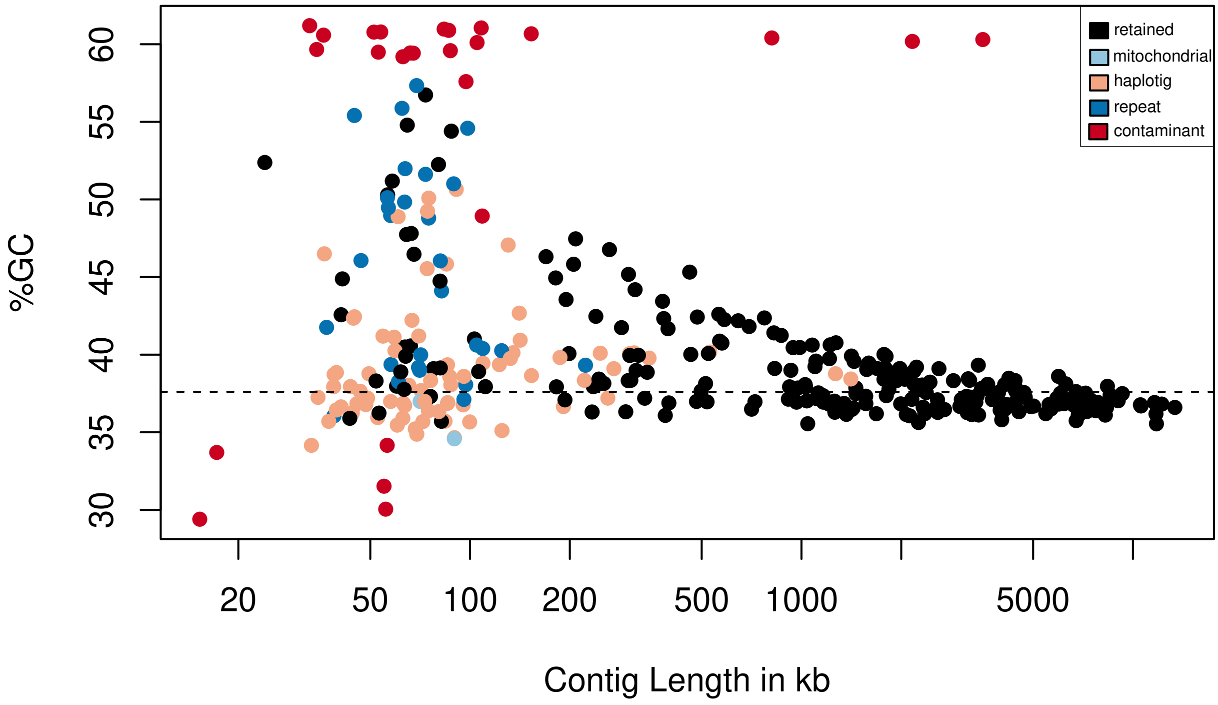

Figure S2: Distribution of the contig sizes of the polished assembly as a function of GC content. The solid line indicates the average GC content. Contigs identified as mitochondrial, contaminant or alternative chromosomal copies (see M&M section of the main text) are identified by different colors.

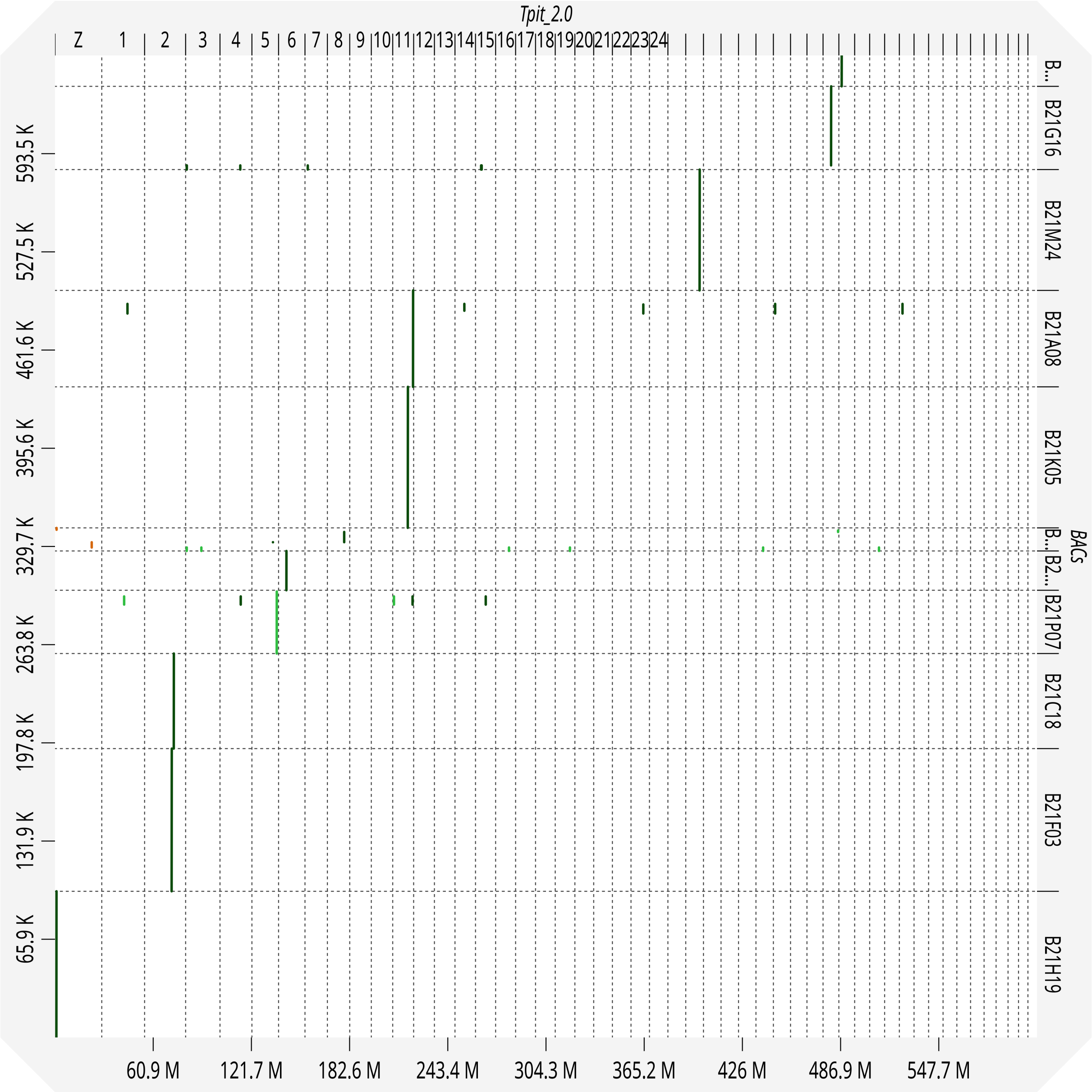

Figure S3: Dotplot generated with `dgenies` (v1.5.0; Cabanettes and Klopp, 2018) comparing the `Tpit_2.0` scaffolded assembly with the sequences of 11 BACs (Gschloessl *et al.*, 2018). Only the largest contig was considered for the 7 BACs with non contiguous sequence.

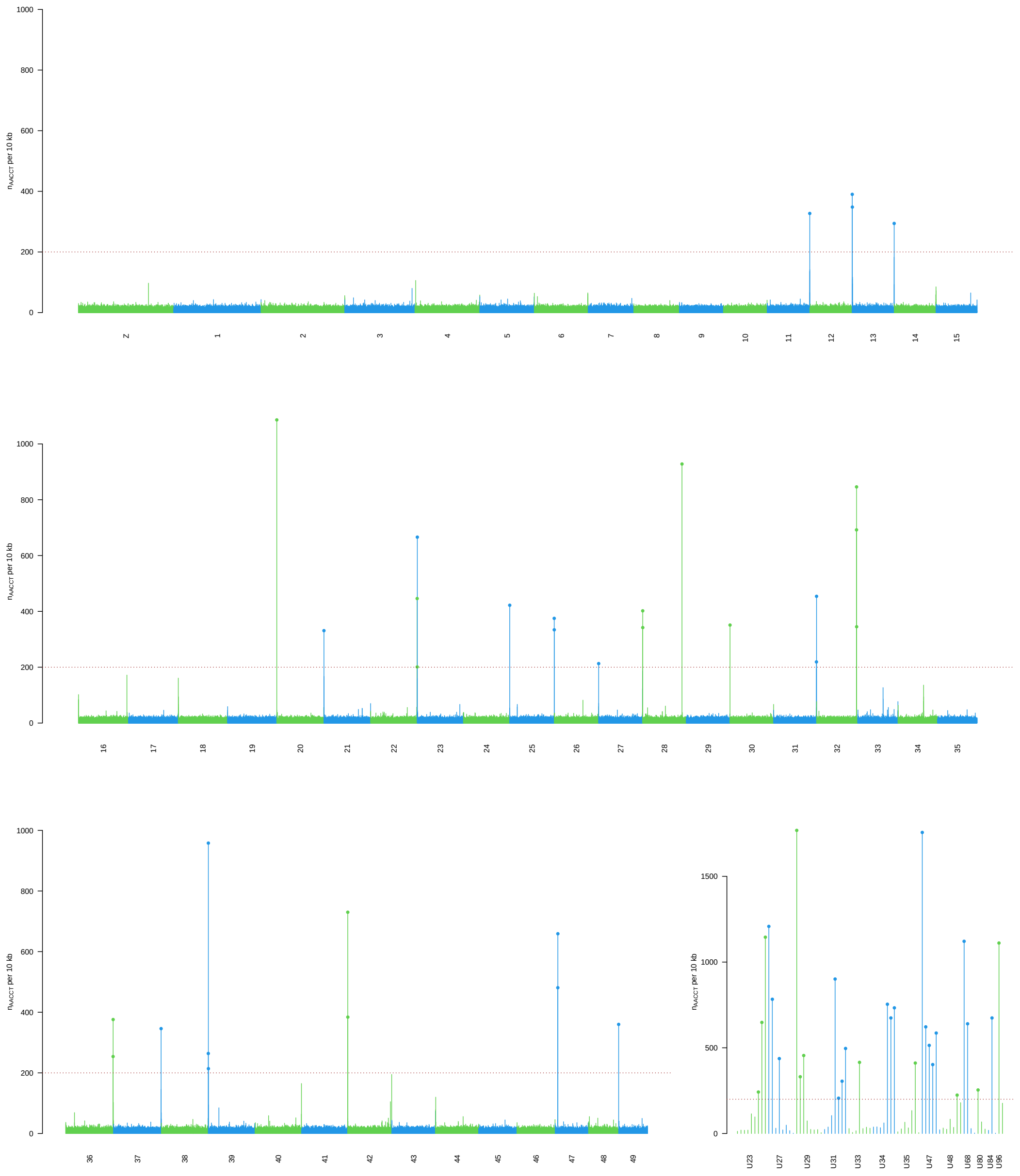

Figure S4: Detection of telomeric motif (AACCT) occurrences across assembled scaffolds. The plot shows the number of AACCT repeats per non-overlapping 10 kb window along the 50 largest chromosome-scale scaffolds (represented with alternating green and blue bars) and 13 smaller scaffolds (¡200 kb) that each contain at least one window with >200 repeats. The horizontal red dotted line indicates the arbitrary threshold of 200 repeats used to define a clear telomeric footprint. Individual windows exceeding this threshold are marked with dots.

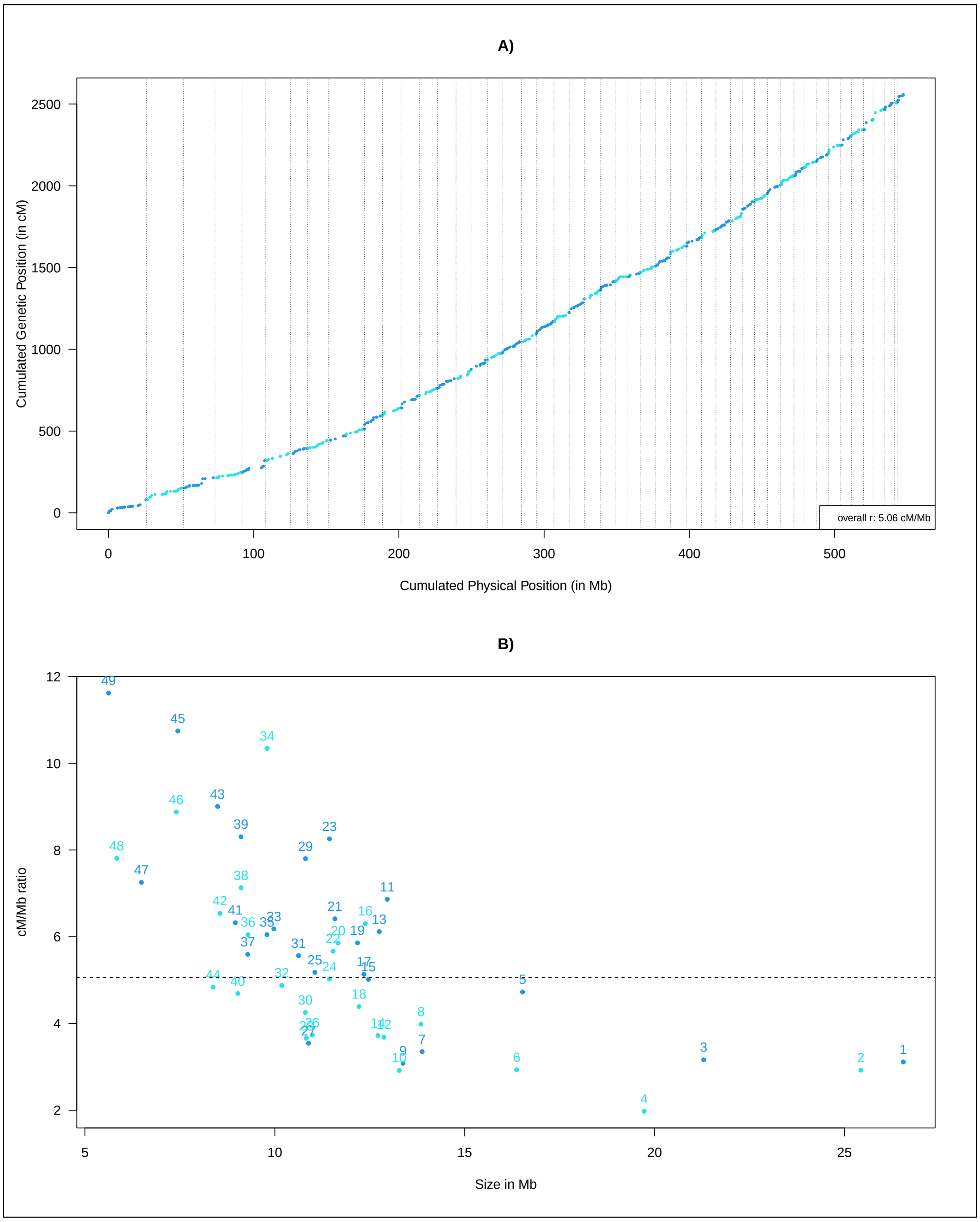

Figure S5: Comparison of the physical and genetic maps of the 49 autosomes. A) Cumulative genetic positions (in centimorgans, cM) of the 672 markers (from collapsed RAD loci; see main text and Table S3) plotted against their cumulative physical positions (in megabases, Mb) along the `Tpit_2.1` genome assembly. Chromosomes are shown in alternating colors and separated by vertical dotted lines. B) Ratio of genetic to physical distance (cM/Mb) for each chromosome as a function of its physical length on the assembly. The horizontal dotted line indicates the genome-wide average ratio. Note that genetic distances were computed according to the order of the assembly, assuming no recombination in females, and using the default Morgan (linear) mapping function as implemented in the `LepMAP3` package (Rastas, 2017).

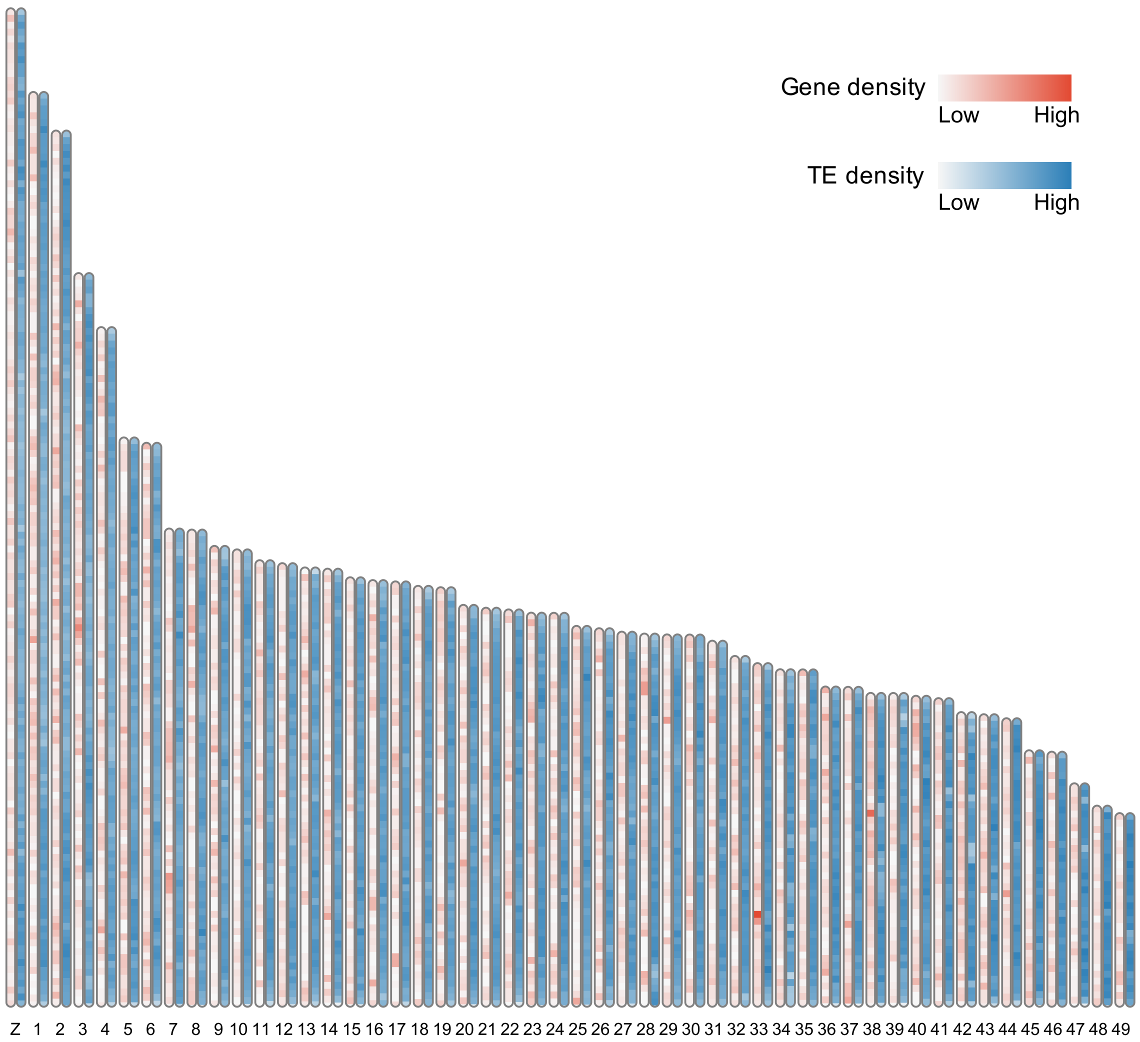

Figure S6: Gene (in red) and transposable element densities (in blue) computed over 200 kb windows along the 50 chromosomes of the *T. pityocampa* assembly.

Supplementary Files

Supplementary File S1. Detailed annotation results and discussion for detoxification genes (pdf format).

Supplementary File S2. Spreadsheet containing annotation results for detoxification genes (OpenDocument format).
